# Fermented grapeseed oil repairs chemically damaged hair via enhanced permeability mechanisms

**DOI:** 10.1038/s41598-025-06571-z

**Published:** 2025-07-01

**Authors:** Nan Wu, Leilei Zhi, Gaixiang Wang, Yu Liu, Jun Feng, Bing You

**Affiliations:** 1 R&D Centre, PeiLai Brand Management Co., Ltd., Building A, Noah Wealth Centre, No.1226 Shenbin South Road, Minhang District, Shanghai, 201106 China; 2 Shanghai WEIPU Testing Technology Group Co., Ltd, No.9 Building, No.135 Guowei Road, Yangpu District, Shanghai, 200438 China

**Keywords:** Hair damaged, Fermented grapeseed oil, Colour resistance, Mechanical properties, Biochemistry, Lipids

## Abstract

Human hair is a biopolymer composed of keratin filaments, lipids, pigments, and water. Chemical treatments of hair, such as dyeing, perming, and bleaching, can impair the integrity of the hair protein structure and accelerate the loss of water and lipids, resulting in increased fragility, breakage, and rapid colour fading. This study investigates compositional changes in fermented grapeseed oil (F-GO) compared to grapeseed oil (GO) and identifies its enhanced hair permeability, highlighting its substantial potential to mitigate hair damage from bleaching and dyeing. The fluorescent labelling results verified that the ability to permeate hair was significantly improved by F-GO compared to GO and silicone oil. Fourier transform infrared spectroscopy (FT-IR) and thermal weight analyser (TGA) results illustrate that F-GO-treated hair can form additional intermolecular hydrogen bonds and might cause a conformational change in the protein structure. The in vitro assessments demonstrate that F-GO delays hair colour loss and enhances mechanical properties. The colour fixation mechanism of F-GO is attributed to its ability to act as an anti-oxygen barrier on the outer layer and a lipid barrier in the inner layer. Together, they protect against pigment loss and degradation. F-GO enhances damaged hair strength by forming hydrogen bonds with keratin residues and preserving lipids and moisture contents. Therefore, F-GO has broad application prospects for bleaching and dyeing damaged hair improvement.

## Introduction

Human hair has a multi-layered structure, the outermost cuticle, the middle cortex, and the innermost medulla. The hair cuticle is essential in controlling of elements into and out of the cortex. It prevents external environmental factors and keeps the hair surface smooth and glossy^1^. The hair cortex is the part contributing most to the mechanical strength of the fibres^2^. Indeed, hair colours are also dependent on natural colour pigments in the cortex. The hair medulla is typically not present in fine hair fibres but becomes more noticeable as the fibre diameter increases, though its contribution to the properties of hair is considered minimal.

The process of enhancing the colour or structure of the hair shaft is known as hair beauty, but it can also lead to damage, both externally and internally, from activities such as combing, perming, dyeing, bleaching, or exposure to other chemicals. McMullen et al. (2020) pointed out that bleaching causes the most damage to the endocuticle and cell membrane complex (CMC), evidenced by erosion of these components in the Atomic Force Microscope (AFM)^3^. Noticeable, CMC, the main structural lipid in hair, acts as the primary adhesive that connects cuticle and cortical cells. The integrity of CMC in hair is susceptible to damage from various environmental and physicochemical factors, leading to the loss, warping, and detachment of hair cuticles. Indeed, the hair bleaching process is the irreversible physicochemical change of melanin under the action of oxidant, forming light-coloured compounds. At the molecular level, hair bleaching allows for disulfide bond breakdown, leading to the formation of cysteine residues^4^. Thus, due to the destructive bleaching procedure, crevices, cracks, and cavities exist in the cortical cells of the bleached hair.

After the bleaching process, which disrupts the cuticle structure to lighten hair, dyeing often follows, with colorants exploiting the already compromised hair shaft to penetrate deeper, exacerbating damage through chemical processes. Hair colorants exhibit divergent mechanisms based on their penetration depth, with permanent (oxidative) and non-permanent (temporary/semi-permanent) dyes differing fundamentally in how they interact with the hair’s structural layers. Temporary and semi-permanent dyes cannot reach the cortex and remain on the surface of the cuticle. The principle of permanent hair dyes can be roughly divided into three steps: expansion, infiltration, and oxidation^5^. The alkaline base disrupts the hair structure to enhance the permeability of the hair cuticle. Colourless precursors enter the cortex, oxidize the hair melanin, and then become large molecules to remain in the hair cortex, under healthy hair conditions, theoretically less likely to diffuse out. However, in damaged hair, common after bleaching or repeated dyeing, the compromised cuticle, and cortex fail to retain pigment molecules effectively, leading to accelerated colour fading, dryness, and mechanical brittleness.

Hair care products frequently incorporate ingredients such as keratin^6^, peptides^7^, amino acids^8^, hyaluronic acid^9^, mineral oils, silicones, and herbal oils. The physical characteristics of hair are primarily determined by its lipid compositions, that consist of free fatty acids, cholesterol, ceramides, cholesterol esters, and cholesterol sulphate^10^. These lipids form a protective barrier in the cuticle, cortex, and medulla of the hair shaft, providing protection against environmental and chemical damage while also influencing the hair’s elasticity and tensile properties^11^. Thus, oil plays a crucial role in safeguarding hair from damage. The penetration capabilities of herbal oils into hair have primarily been studied with a focus on coconut oil, which has been asserted that oil penetration had a beneficial effect on hair strength^12–16^. Considerable variability in hair shaft permeability has been documented across distinct oil classes, primarily governed by their molecular architecture (e.g. saturation level, chain length). The mineral oil and the oil have a bulky structure, limiting their penetration of the hair shaft, preventing them from reaching the cortex. Instead, they adhere to the cuticle’s surface, enhancing shine, diminishing friction, and avoiding hair damage. Takahashi et al. (2014) found that unsaturated fatty acids are more present in the cortex of the hair than on the hair’s surface^17^, indicating that external oil supplementation alone may not be sufficient to replenish lost lipids in the cortical layer. The oil with low molecular weight and a straight linear chain, is capable of penetrating the hair shaft and filling the gaps between cuticle cells^18,19^. Then it can prevent the penetration of aggressive substances such as surfactants into the follicle and reduce the amount of water absorbed in the hair, leading to a lowering of swelling^20^.

Since the inherent structure of natural oils presents significant challenges for modification, a more accessible approach to enhancing hair penetration efficiency involves the fermentation process. This study explores the permeation characteristics of grapeseed oil (GO), both prior to and following fermentation, utilising fluorescent labelling techniques for analysis. Grapeseed oil was selected due to its notable antioxidant properties, proven efficacy in hair care, and favorable cost-performance ratio^21^. However, the existing literature lacks comprehensive insights into the penetrability of GO into hair fibres. There has yet to be a systematic investigation that links the penetration efficiency of fermented oils with their functional properties and underlying mechanisms. The current paper investigated the potential underlying mechanisms explored through FT-IR, TGA, and BET characterization of hair treated with fermented grapeseed oil (F-GO). Furthermore, this study assesses the efficacy of F-GO as a potential therapeutic agent for hair damage resulting from bleaching and dyeing processes, which aims to establish a foundational framework and offer innovative perspectives on hair damage recovery.

## Results

### Identification of grapeseed oil before and after fermentation

 Grapeseed oil contains high levels of hydrophilic constituents, including a large number of phenolic compounds, and lipophilic constituents such as vitamin E, unsaturated fatty acids (UFAs), and phytosterols. In terms of fatty acid (FA) composition, linoleic acid (C_18:2_) is the most abundant FA in grapeseed oil, followed by oleic acid (C_18:1_) and palmitic acid (C_16:0_)^22^. Triglycerides (TAGs), is formed by combining one glycerol molecule with three fatty acid molecules and is a major kind of oil components. Ultra-high performance liquid chromatography-tandem triple quadruple mass spectrometry (UHPLC-MS/MS) was employed to identify the TAGs in both GO and F-GO. Due to the predominance of 16 and 18-carbon chain lengths in the fatty acids of grapeseed oil, our analysis focused on changes in TAGs of 52 and 54 total carbon chain lengths. As depicted in Table 1, among TAGs with identical carbon chain lengths, the concentrations of TAGs exhibiting a lower degree of unsaturation tended to decline following fermentation (e.g., TAGs(52:3), TAGs(54:3), TAGs(54:4)), while the content of higher unsaturated TAGs groups appeared to increase (e.g., TAGs(52:4), TAGs(54:5), TAGs(54:6) and TAGs(54:7)).

**Table 1:** List of TAGs (C_52_ and C_54_) detected in GO and F-GO by UHPLC-MS/MS.

Types of TAGs		F-GO/%	GO/%	D-value/%
TAGs (52:1)	TAG (52:1 FA18:0)	0.35	0.36	− 0.01
	TAG (52:1 FA18:1)			
	TAG (52:1 FA16:0)			
TAGs (52:2)	TAG (52:2 FA18:0)	2.86	2.94	− 0.08
	TAG (52:2 FA18:1)			
	TAG (52:2 FA18:2)			
	TAG (52:2 FA16:0)			
TAGs (52:3)	TAG (52:3 FA18:1)	8.10	8.81	-0.7
	TAG (52:3 FA18:2)			
	TAG (52:3 FA16:0)			
TAGs (52:4)	TAG (52:4 FA18:1)	16.60	16.12	0.48
	TAG (52:4 FA18:2)			
	TAG (52:4 FA18:3)			
	TAG (52:4 FA16:0)			
TAGs (52:5)	TAG (52:5 FA16:1)	0.39	0.40	− 0.01
	TAG (52:5 FA18:2)			
TAGs (54:1)	TAG (54:1 FA18:0)	0.04	0.05	− 0.01
TAGs (54:2)	TAG (54:2 FA18:0)	0.60	0.61	− 0.01
	TAG (54:2 FA18:1)			
	TAG (54:2 FA18:2)			
TAGs (54:3)	TAG (54:3 FA18:0)	4.37	4.58	− 0.21
	TAG (54:3 FA18:1)			
	TAG (54:3 FA18:2)			
TAGs (54:4)	TAG (54:4 FA18:0)	14.13	14.42	− 0.29
	TAG (54:4 FA18:1)			
	TAG (54:4 FA18:2)			
	TAG (54:4 FA18:3)			
TAGs (54:5)	TAG (54:5 FA18:1)	19.49	19.24	0.25
	TAG (54:5 FA18:2)			
	TAG (54:5 FA18:3)			
TAGs (54:6)	TAG (54:6 FA18:1)	31.62	31.16	0.46
	TAG (54:6 FA18:2)			
	TAG (54:6 FA18:3)			
TAGs (54:7)	TAG (54:7 FA18:2)	1.42	1.31	0.11
	TAG (54:7 FA18:3)			

The above data are the means of the triplicate experiments. D-value is the content of F-GO minus that of GO.

**Fig. 1:**
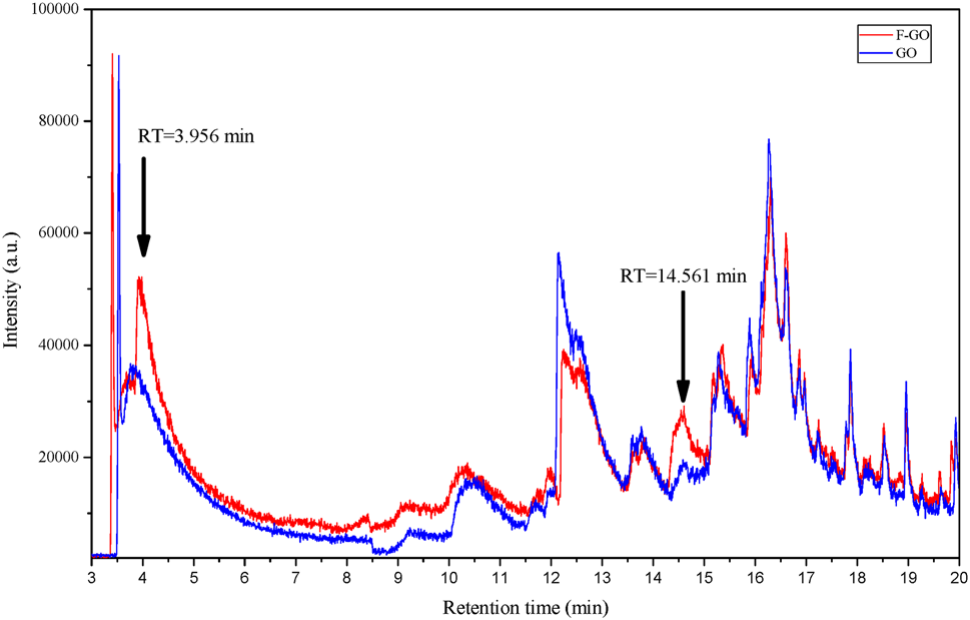
Total ion chromatogram (TIC) of F-GO and GO showing intensity variations at different retention times.

This study focuses on the differences in volatile and semi-volatile substances below 305 °C between GO and F-GO. By comparing the gas chromatography-mass spectrometry (GC-MS) chromatograms of grapeseed oil before and after fermentation, it was found that the total ion current (TIC) of the GC-MS of F-GO is more complex than that of GO, as evident from the data in Fig. 1, indicating that the fermentation process promotes the generation of small molecule volatile and semi-volatile substances. Specifically, the contents of substances at retention times (RT) of 3.956 min and 14.561 min in F-GO increased significantly. Combined with the analysis of mass spectra, it is preliminarily speculated that the substances at these two retention times might be ethanol and phenylethanol, respectively. These substances may be produced through a series of biochemical reactions during fermentation, thus altering the composition of grapeseed oil.

### Penetration of fluorescence labelled F-GO into hair fibres

**Fig. 2:**
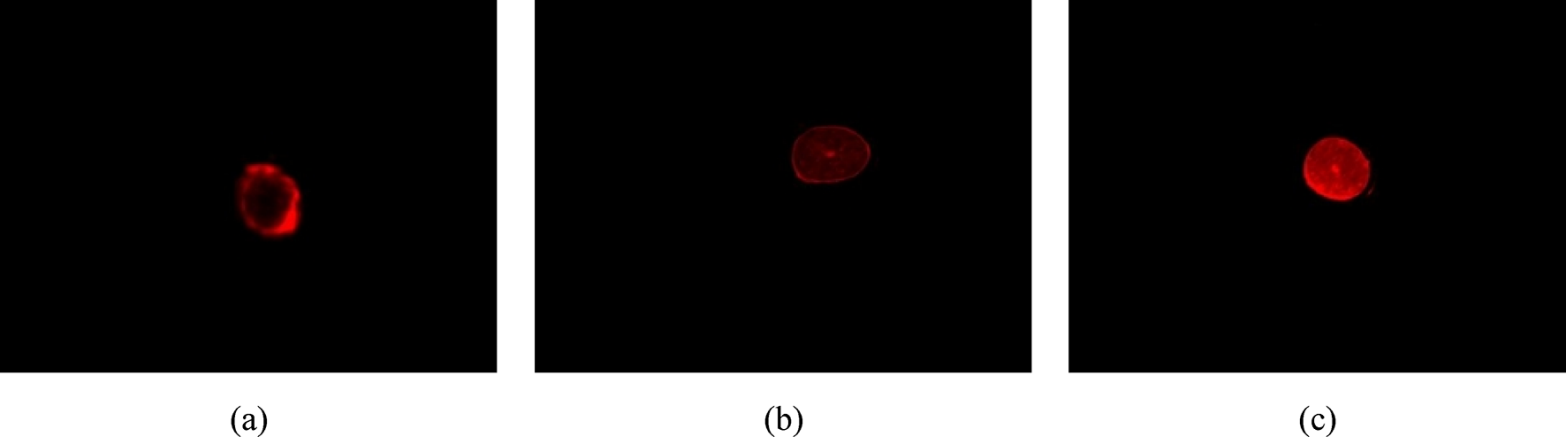
Cross sections of hair treated with fluorescent-labelled silicone oil (**a**), GO (**b**) and F-GO (**c**).

 The fluorescence microscopy was used as a sensitive technique to investigate the differences in the penetration behaviour of oil with diverse properties. Cross-sectional images of hairs treated with fluorescently labelled silicone oil and grapeseed oil before and after fermentation, as depicted in Fig. 2. The results demonstrated that grapeseed oil exhibited superior permeability, penetrating all layers of the hair, whereas silicone oil only reached the outermost layers of the fibres. Moreover, the fluorescence intensity of hair treated with F-GO was notably higher than that of hair treated with GO. Specifically, the average fluorescence intensities at the hair cross-section for silicone oil, GO, and F-GO were 6.554, 13.475, and 35.854, respectively. Similar trends were also observed in the area percentages of permeated substances in hair cross-sections, with values of 16.578%, 39.401% and 86.948% for the silicone oil, GO, and F-GO treatment groups, respectively. The images in Fig. 2 and the aforementioned data indicate that the permeation efficiency of silicone oil into hair is far lower than that of GO and F-GO, with silicone oil only adhering to the outermost layer of the hair. In contrast, both F-GO and GO can penetrate into the inner hair structure. Notably, the permeation efficiency of F-GO shows a statistically significant enhancement compared to GO, which means fermentation significantly enhances the permeation efficiency of grapeseed oil into hair.

### FT-IR characterization of the F-GO-treated hair

**Fig. 3:**
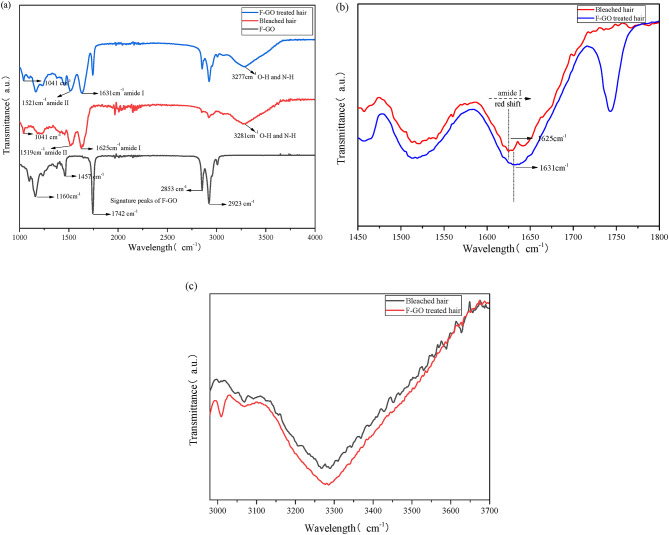
Comparison of the infrared spectrum of fermented grapeseed oil (F-GO), F-GO treated and untreated bleached hair (**a**); Enlargements of the amide I and II regions in the spectra (**b**); Enlargements of the O-H and N-H stretching-related bands in the spectra (**c**).

 Figure 3a indicates the FT-IR spectra of the F-GO and the bleached hair before and after being treated with F-GO, which were plotted based on the mean values of three repeated experiments after normalization. The alkaline chemical reagents turn the hair surface protein cystine into a cysteine residue, and then the oxidation of hair proteins can be detected from bleached hair, at 1040 cm^− 1^, which is regarded as a characteristic peak of cystine oxidation peak^23^. The two rather prominent peaks centred near the 1480–1690 cm^− 1^ band are the most obvious section of the characteristic peaks in the infrared spectrum of protein-peptide chains, mainly described as the deformed vibration of the-CONH-group of amide I/II in the protein structure^24^. The absorption peak in the amide I zone at 1620 cm^− 1^ is mainly attributed to the α-helix, while the amide II band at 1520 cm^− 1^, associated with the N-H bending vibration and C-N stretching vibration. Upon magnification of the amide I and amide II regions in Fig. 3(b), peak splitting in the amide I band of untreated bleached hair is observed, likely attributed to conformational changes in keratin induced by bleaching^25^. Increased structural disorder introduces variations in amide I vibration frequencies, manifesting as a double peak. Following treatment with F-GO, this splitting phenomenon disappears, and the amide I peak red-shifts from 1625 cm^− 1^ to 1631 cm^− 1^. Such variation in the amide I bands might be associated with F-GO penetration and the formation of intermolecular H-bonds with keratin residues in hair. It further indicates that F-GO treatment may induce a conformational change in the protein structure, shifting from a looser to a tighter state. The spectra of both bleached hair samples showed a broad band at 3100 to 3300 cm^− 1^ speculation arises from the O-H and N-H stretching-related bands in the hair, the intensity of which was increased and the peak shape was wider after the F-GO treatment, as shown in Fig. 3(c), which also could be explained by the enhancement of intermolecular hydrogen bond.

### Thermal properties of hair treated with F-GO

**Fig. 4:**
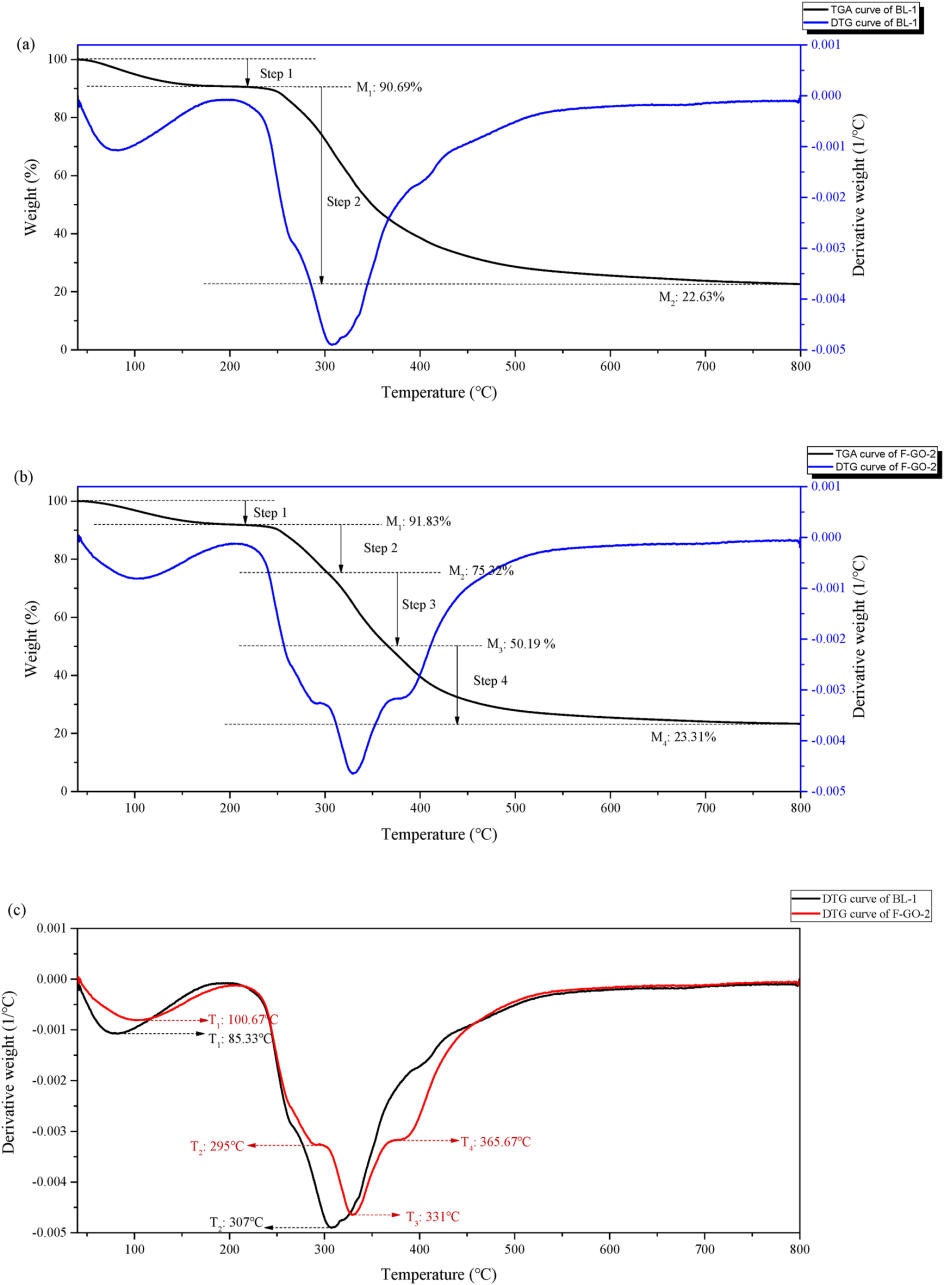
The TGA/DTG (derivative thermogravimetry) curves of BL-1 (**a**) and F-GO-2 (**b**) with Dual Y-Axes: weight (%) and derivative weight (1/°C) vs. Temperature (°C); The DTG curves of BL-1 and F-GO-2 (**c**).

 Thermogravimetry is a highly effective technique for elucidating the putative mechanisms of solid-state reactions, such as thermal decomposition and dehydration. The initial mass loss observed in hair samples is due to the release of water, which happens between 25 °C and 170 °C. Subsequent mass loss stages occur around 250 °C and 330 °C and are related to the denaturation of hair keratin^26^. This process involves the organic degradation of the hair microfibrils and matrix. Table 2 presents the TGA results of bleached hair (BL, untreated with F-GO) and F-GO-treated hair, including inflection point temperatures (°C) and residual mass (%). Three repeated experiments for each sample are listed to showcase data consistency. The thermal decomposition behavior of F-GO-treated hair was compared with the blank (BL) group using TGA/DTG curves (Fig. 4a–c). The F-GO-2 exhibited four distinct inflection points at T₁=100.67 °C, T₂=295 °C, T₃=331 °C, and T₄=365.67 °C, reflecting a more complex decomposition process compared to the BL-1, which showed two major peaks at T₁=85.33 °C and T₂=307 °C (Fig. 4a,b). When overlaid DTG curves in a plot (Fig. 4c), the F-GO-treated hair demonstrated a higher initial dehydration temperature (100.67 °C vs. 85.33 °C for BL), accompanied by a lower water loss rate, indicating improved moisture retention due to F-GO treatment. The keratin decomposition peak, corresponding to the main thermal degradation stage, shifted significantly from 307°C (BL) to 331°C (F-GO). These results collectively indicate that F-GO treatment modulates the thermal degradation pathway of hair, delaying both water evaporation and keratin decomposition, thus improving overall thermal resistance.

**Table 2:** TGA results: inflected point temperature (°C) and residual mass (%) of before and after F-GO treated hair under a nitrogen atmosphere.

Samples		T_1_ / °C	M_1_/%	T_2_ / °C	M_2_/%	T_3_ / °C	M_3_/%	T_4_/°C	M_4_/%
BL	1	85.33	90.69	307.00	22.63	/	/	/	/
	2	87.00	91.23	326.67	28.33	/	/	/	/
	3	82.33	91.34	308.67	26.46	/	/	/	/
F-GO treated	1	100.67	91.38	294.33	73.65	330.00	49.70	363.33	21.83
	2	100.67	91.83	295.00	75.32	331.00	50.19	365.67	23.31
	3	92.33	91.59	299.67	72.89	333.00	50.33	356.21	19.77

Sample BL = Bleached hair without F-GO treatment, T_1_ and T_2_ = inflection temperatures, M_1_ and M_2_ = residual mass of the stage.

### Hair cortex cavities changes with F-GO treatment

**Fig. 5:**
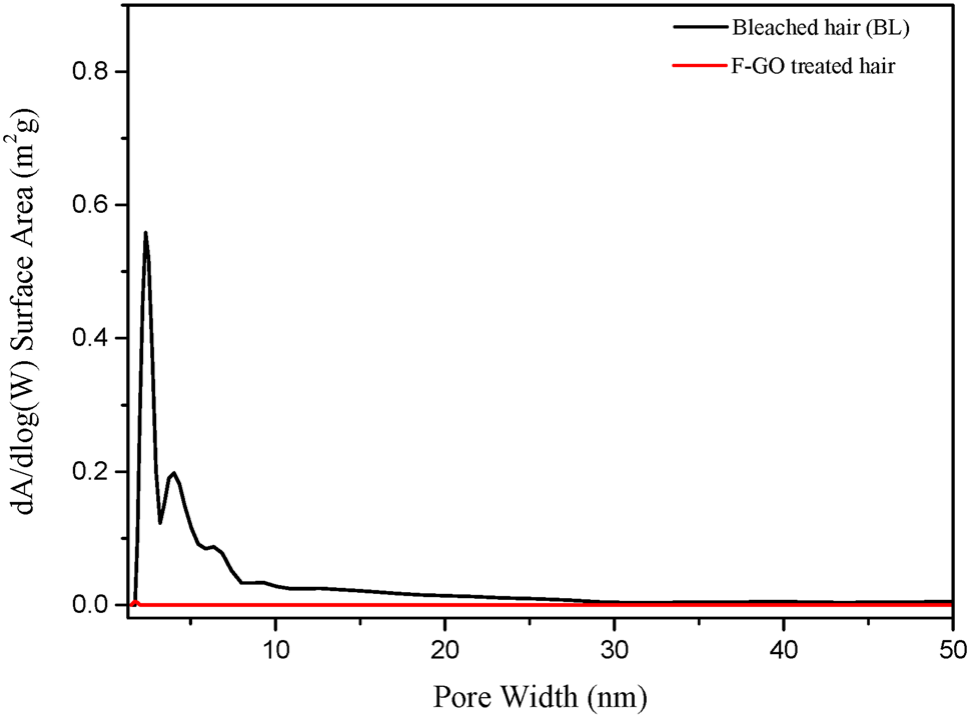
BET analysis of hair pore size distribution.

 Nitrogen sorption measurements, by the Brunauer-Emmett-Teller (BET) theory, quantitatively assess the changes in pore structure within the hair cortex^15^. The migration of surfactant molecules from the endocuticle to the cortex increased porosity due to the solubilization of protein segments. Examination of the pore size distribution map (as shown in Fig. 5) indicates a specific number of apertures ranging from 0.5 to 10 nm after treatment with SLES. Notably, the peak at 0.5–10 nm disappears after applying F-GO. F-GO molecules fill the pores in the hair cortex so as to reduce the porosity of the hair fibres.

### Effects of F-GO treatments on mechanical properties of bleached hair

 Minor damaged hair bundles were treated with F-GO, as described in the experimental section. Table 3 shows the mechanical properties of the bleached hair before and after F-GO treatment, with a statistically significant improvement. The break load increased by 18.73%, and the total work rose by 30.77%. It demonstrates that F-GO could highly enhance the mechanical properties of damaged hair.

**Table 3:** Effects of F-GO treatments on tensile strength.

Parameters	Group	Statistical items							
		N	Average	STDEV	Median	STDEVP	MIN	MAX	*P* value
Break Load/gmf	BL	30	102.0	3.573	98.08	19.57	68.85	136.6	< 0.01^**^
	F-GO treated	30	121.1	5.421	114.2	29.69	75.40	183.1	
Total Work/J	BL	30	0.0013	0.0001	0.0014	0.0003	0.0009	0.0020	< 0.01^**^
	F-GO treated	30	0.0017	0.0001	0.0016	0.0005	0.0009	0.0030	

Group BL = bleached hair without F-GO treatment.

“ns” represents not significant; “*” represents *P* < 0.05; “**” indicates *P* < 0.01; and “***” indicates *P* < 0.001.

### Colour protection by F-GO

**Fig. 6:**
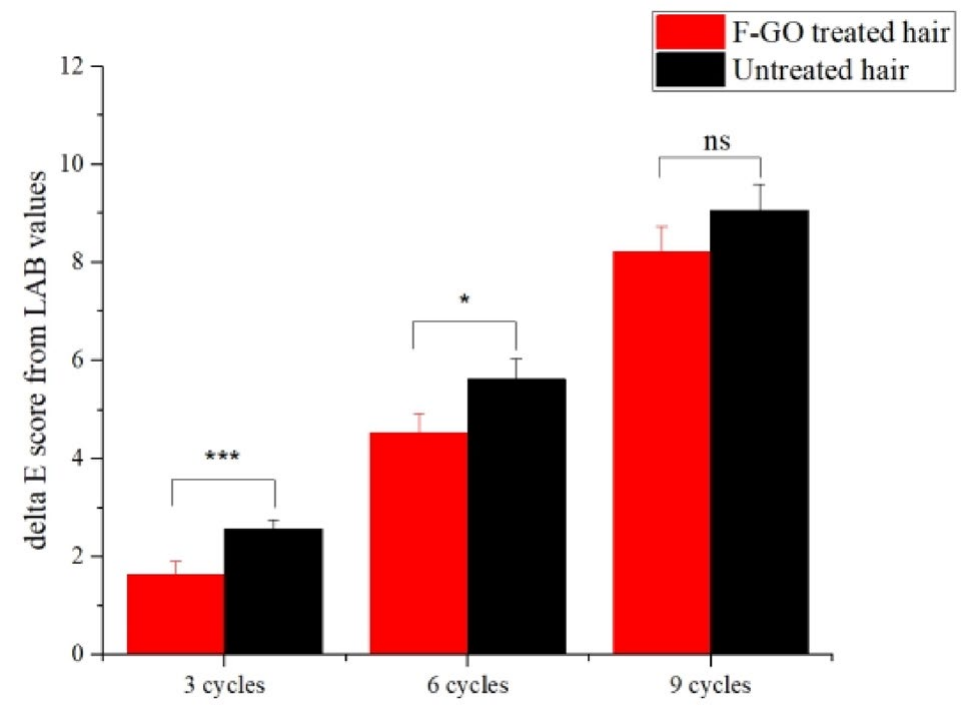
Average ∆E computed from the coloured control tress (*n* = 3). “ns” represents not significant; “*” represents *P* < 0.05; “**” indicates *P* < 0.01; and “***” indicates *P* < 0.001.

 The quantitative measurement of hair colour was conducted with a spectrophotometer, the ∆E plot displayed in Fig. 6. The loss of hair colour occurs at a slower rate after F-GO treatment. However, the difference decreases with the number of wash cycles, although it still demonstrates the strong hair colour-resistant effect of the F-GO.

## Discussion

Several plant oils with diverse TAG compositions have demonstrated the ability to penetrate hair^27^. Marsh et al. (2024) studied the penetration ability of TAGs present in oil with different chain lengths and degrees of saturation, indicating a correlation between enhanced penetration efficacy, shorter chain lengths, and higher presence of unsaturation in the fatty acid chains^28^. Table 1 previously showed increased unsaturation in C_52_-C_54_ TAGs post-fermentation through UHPLC-MS/MS, suggesting a possible connection to enhanced hair permeability. Oil fermentation may generate additional polar metabolites, such as small-molecule alcohols, which also could contribute to the enhanced hair permeability of fermented oils. Through GC-MS, we preliminarily determined that alcohols (e.g. ethanol and phenylethyl alcohol) might be generated during the fermentation process. It is speculated that the small molecule alcohols produced by fermentation may help F-GO penetrate into the hair cortex more easily by temporarily opening the hair cuticles and causing lipid perturbation. Oil fermentation may also generate other substances that facilitate the penetration of oils into hair. Further exploration of the changes in oil fermentation composition represents a critical research direction aiming to elucidate the functional mechanisms of fermented oils.

Hair cross-sectional fluorescence microscopic images were analyzed using ImageJ software with semi-quantitative methods to quantify the permeation efficiency of different oils. Analysis showed that the average fluorescence intensity of F-GO exhibited 5.47 folds and 2.06 folds significantly higher hair permeation efficiency compared to silicon oil and GO, respectively. Then, the internal chemical composition and thermal stability of damaged hair treated with F-GO evaluated through FT-IR and TGA analyses, thereby providing an indirect understanding of the interconnections within the hair structure. Both experiments were selected to bleach damaged hair bundles, as bleaching oxidizes the existing melanin in the cortex. The bleaching process, which oxidizes melanin in the cortex, breaks down disulfide bonds in keratin and may compromise the cuticle structure (more porous)^29^. Treatment of bleached hair with F-GO induced the formation of intermolecular hydrogen bonds between keratin amino acid residues and F-GO, as evidenced by FT-IR spectroscopy (Fig. 3a–c). Specifically, the peak splitting in the amide I band disappeared, accompanied by a red-shift (1625 to 1631 cm⁻¹), indicating that keratin structure transitioned from a loose, disordered state to a more ordered, homogeneous conformation. This structural rearrangement was further corroborated by thermogravimetric analysis (TGA), F-GO-treated hair exhibited a more complex decomposition profile, with delayed water evaporation and keratin degradation compared to the control group (Table 2; Fig. 4(a-c)). The mechanical properties and structure of hair are often linked to the presence of hydrogen, disulfide, and ionic bonds within the keratin fibres. Breakspear et al. (2024) have hypothesized an estimated ratio of nine hydrogen bonds to one disulfide bond within these fibres. The presence of hydrogen bonds significantly influences the mechanical properties of hair fibres^30^. As a result, the formation of intermolecular hydrogen bonds with F-GO may contribute to enhanced mechanical strength in damaged hair, as suggested by the data in Table 3.

The lipid barrier is crucial in preventing the infiltration of external substances (such as surfactants) and the loss of internal pigments and moisture. An increase in fibre porosity caused by the solubilization of protein segments can also lead to an increase in the diffusivity of the fibre. As illustrated in Fig. 5, F-GO introduced into the fibre structure can reduce the porosity of hair fibres, may also prevent surfactant access to proteins, thus limiting the formation of new pores. Based on the results from Fig. 6, after nine cycles of washing, F-GO proves effective in reducing pigment loss in hair, thus preserving its colour.

Based on the assessment techniques carried out, we hypothesize the mode of action of F-GO on hair classified under the below sites of action:

1. Hair cuticle protective layer (on the surface): The formation of an external occlusive layer on the hair surface using F-GO effectively protects the hair from exposure to ultraviolet (UV) light, water, and surfactants, owing to the remarkable anti-oxidant and hydrophobic properties of F-GO. The photochemical damage to hair encompasses the degradation and depletion of hair proteins and the breakdown of hair pigment^31^. Surfactants utilised during regular hair washing also have a tremendous impact on lipid removal from hair, particularly in cases of severely damaged hair^32^. Grapeseed oil is rich in phenolic compounds, including flavonoids, carotenoids, phenolic acids, tannins, and stilbenes. The primary bioactive property of phenolic compounds lies in their antioxidative potential^22^. As a result, F-GO covers with the hair cuticle to creates an outer barrier, preserving the structural integrity of the hair shaft by mitigating the exposure to UV radiation, moisture, and surface-active agents.
2. Enhanced hair permeation efficiency: Two key factors as fermentation increases the proportion of highly unsaturated triacylglycerols (TAGs, C_52_-C_54_) in grapeseed oil, and generates small-molecule alcohols, which likely contribute to its superior penetration compared to GO. Penetration of the individual pure TAGs was confirmed higher penetration for shorter chain lengths and unsaturated fatty acids^28^. While direct evidence of alcohols promoting hair shaft penetration is currently lacking, this hypothesis is biologically plausible based on their established roles in enhancing skin and follicular permeation^33–35^.
3. Cavities replenishment (in the core): F-GO penetrates and progressively fills the cavities inside the damaged hair core, which forms a dense lipid barrier. Thus, strengthening the lipid components packing structure inside the hair can result into better resistance towards leaching out and maintaining the structure against the surfactant assault.
4. Keratin conformation optimization and hydrogen bond formation (in the core): F-GO treatment is hypothesized to mitigate bleaching-induced damage by facilitating intermolecular hydrogen bond formation between the oxygen-containing functional groups from highly unsaturated TAGs in F-GO, and polar amino acid residues in keratin. Further mechanistic studies will be essential to resolve the specific hydrogen bond partners and structural rearrangements induced by F-GO, providing a molecular-level understanding of its protective effects against hair damage.

Overall, this study advances the theoretical basis for using fermented grapeseed oil in hair care, highlighting fermentation as a viable strategy to develop next generation lipid-based hair care ingredients with enhanced penetration and protective efficacy.

## Methods

### Materials

 Minor damaged human hair bundles from CANYU (Dark brown, 17 cm·1.5 cm·2.5 g, China), Severely damaged human hair bundles from CANYU (Golden yellow, 17 cm·1.5 cm·2.5 g, China), Sodium Lauryl Ether Sulphate (SLES, ‌Guaranteed Reagent, Sinopharm, China), Methanol (MeOH, ‌Guaranteed Reagent, Anpel, China), Ammonium formate (NH_4_Ac, ‌Guaranteed Reagent, Hushi, China), Acetonitrile (ACN, Guaranteed Reagent, Anpel, China), Isopropanol (IPA, Guaranteed Reagent, Anpel, China), Stain and developer (Jihoda, China), Ultra High Performance Liquid Chromatography (Nexera UHPLC LC-30 A, Shimadzu, Japan), Triple Quadrupole Mass Spectrometry (AB Sciex 5500, SCIEX, America), Integrated Fluorescence Microscopy Imaging System (BZ-X810, Keyence, Japan), Fourier Transform Infrared Spectrometer (Nicolet iS10, Thermo Fisher Scientific, America), MultiPurpose Sampler (MPS, GERSTEL, Germany), Gas chromatography-mass spectrometry (7890B-5977B MSD, Agilent, America), Chromatographic column (TG-5SilMS, 60 m×0.25 mm×0.25 μm, Thermo Fisher Scientific, America), Freezing Microtome (CM1950, LEICA Biosystems, Germany), Thermos Gravimetric Analysis (TGA2, Mettler Toledo, Switzerland), Surface Area and Porosity Analyzer (ASAP2460, Micromeritics, America), Chroma Meter (CR400, Konica Minolta, Japan).

### Fermentation of grapeseed oil

 200 mL of water autoclaved, cooled to room temperature, add *Candida lipolytica* strain, mixed well, then add 1000 g of grapeseed oil, 30°C with shaker, fermentation at 150 rpm, for 4 to 6 days. After that, stand for 3–6 h, add white diatomite for filtration, and finally take the clear liquid.

### Characterization of grapeseed oil before and after fermentation

 UHPLC-MS/MS analyses were performed on an Ultra High Performance Liquid Chromatography (Nexera UHPLC LC-30 A, Shimdzu, Japan) and a Triple Quadrupole Mass Spectrometry (AB Sciex 5500, SCIEX, America). Each sample (1µL aliquot) was injected directly into a Phenomenex Kenetic C18 2.6 μm 2.1 × 100 mm (00D-4462-AN). The mobile phase of the analytical HPLC consisted of phase A (MeOH: ACN: H_2_O = 1:1:1, v/v, 5mM NH_4_Ac) and phase B (IPA with 5mM NH_4_Ac). The gradient program was set as follows: 20-40% B (0.5–1 min), 40-60% B (1.5–3 min), 60-98% B (3–13 min), 98%-20% B (13–13.1 min), 20%-20% B(13.1–17 min). Column temperature was at 45 °C, and the tray temperature was maintained at 15 °C. All LC-MS grade solvents were purchased from Anpel. The buffer of 5mM ammonium formate (in water) was prepared with the chemical from Hushi. The GC-MS analysis was performed using an Agilent 7890B gas chromatograph coupled with a 5977B Mass Selective Detector (MSD). A TG -5silms capillary column with dimensions of 60 m×0.25 mm×0.25 μm was used for the separation of compounds. A volume of 3 mL of the detected sample (GO/F-GO) was accurately measured and transferred into separate solid-phase microextraction vials. The vials were then tightly capped to prevent any loss of volatile components and were ready for subsequent instrumental analysis. A split ratio of 10:1 was used, which was optimized to ensure proper sample introduction and chromatographic separation. The scan range of the mass spectrometer was set from 29 to 550 amu, covering a broad range of possible molecular weights to detect various compounds in the samples. The initial temperature of the column was maintained at 40 °C for 5 min to allow for the proper focusing of the analytes. Then, the temperature was increased at a rate of 15 °C per minute to 280 °C and held for 5 min. Subsequently, the temperature was further increased at the same rate to 305 °C and held for another 5 min. The inlet temperature was set at 270 °C to ensure complete vaporization of the injected samples. The column flow rate of the carrier gas was maintained at 2 mL·min^− 1^. Helium was used as the carrier gas due to its inertness and good separation performance.

### Fluorescent labelling and fluorescence microscopy

 Three severely damaged hair bundles were taken from the same batch, and 1mL of 10% SLES was used for basic cleaning before testing. The hair bundles were hung in a constant temperature and humidity environment for drying and balance. The test oil and Nile red fluorescent marker were weighed in test tubes, then seal to dissolve 5 mg·L^− 1^ F-GO-Nile red solution. The hair was immersed in the stained fluorescent solution, soaked for 8 h before removing and wiping the hair surface. Finally, hair cross-sections were obtained by using frozen sections on two randomly selected hairs and observing the cross-sections using a fluorescence microscope. This experimental manipulation was repeated for two other samples treated with grapeseed oil and silicone oil. Fluorescence microscopic images of hair cross-sections were analyzed using Image J software to assess the permeation efficiency of different oils. This analysis involved quantifying two key parameters: average fluorescence intensity within the hair cortex and the area percentage of permeated substances, both serving as semi-quantitative indicators for comparative evaluation.

### Characterization of hair

 Six severely damaged hair tresses were randomly divided into two groups: a BL and an F-GO group (three tresses per group). All tresses were pre-washed with 1 mL of 10% SLES prior to testing. The F-GO group was soaked in fermented grapeseed oil for 8 h, while the control group remained untreated. Post-treatment, tresses were air-dried and equilibrated for 24 h in a thermostatic-hygrostatic environment (26 ± 2 °C, 60 ± 10% relative humidity), then gently blotted with dry tissue paper to remove surface residues. An FT-IR spectrophotometer detected hair fibres to obtain spectra ranging from 500 to 4000 cm^− 1^. Blank background profiles were tested and saved. After spreading the sample on the diamond crystal and tightening the upper end pressure rod to ensure that the sample was in close contact with the crystal in ATR mode.

### Thermogravimetric analysis

 Consistent with the hair treatment in the above mentioned FT-IR experiments. Thermogravimetric analysis was performed with a TGA instrument (TGA2, Mettler Toledo, Switzerland). Approximately 5 mg of cut hairs was packed into an aluminium pierced pan (100 µL) and heated under a nitrogen stream from 40 to 800°C at a heating rate of 20°C·min^− 1^. Cooling down from 800°C to 500°C at a rate of 20°C·min^− 1^. After completion, the remaining residue was heated in 500–800°C air atmosphere.

### BET surface area measurement

 The study involved six severely damaged hair bundles, randomly divided into two groups: three bundles in the control group and three in the sample group (treated with fermented grapeseed oil). Each group was treated with 1 mL of 10% SLES before testing. The sample group was soaked in fermented grape seed oil for 8 h, while the control group was not treated. After treatment, the hair bundles were dried and placed in an environment with a constant temperature of 26 ± 2◦C and humidity of 60 ± 10% RH for 24 h. Then, the hair bundles were gently wiped and pressed with a dry towel to remove any residual samples on the surface. Porosity measurements were performed using the Surface Area and Porosity Analyzer.

### Hair tensile property tests

 Minor damaged hair strands were washed with 1 ml 10% SLES solution and naturally dried at ambient conditions (Temperature = 26 ± 2◦C, RH = 60 ± 10%). Different hair strands were treated with 0.5 g F-GO (F-GO mass and hair mass 1:5) and rubbed for a minute. The treated hair strands were stored at ambient conditions for 24 h, free from flushing. The hair strands treated with nothing were set as the negative control group (bleached hair group). 30 hair fibres were selected from the two groups of hair bundles, and the mechanical properties of hair stretching were tested with a hair multi-function tester. The statistical properties of the sample group and the control group were analysed by TTEST, and the test level was α = 0.05, which was significantly different from the results of the control group.

### Hair colour treatment and measurement

**Fig. 7:**
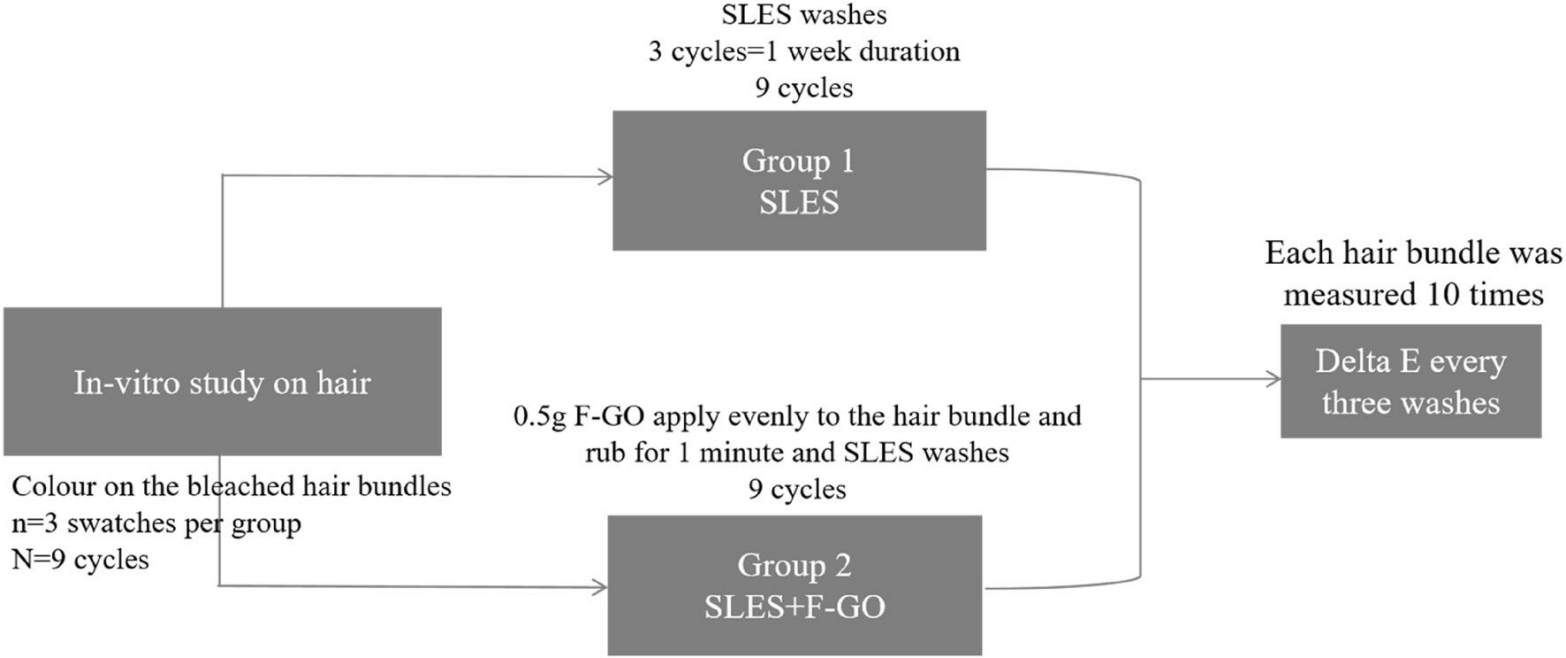
Experimental plan for the treatment of coloured hair bundles.

 The overall scheme for this study is presented in Fig. 7. Prepare the stain and developer mixture in a glass beaker at a 1:1 ratio, ensuring thorough mixing for 1 min. Apply the mixture to a dose of 2.5 g and position it on the test bench (covered with plastic wrap) at room temperature for 30 min. Subsequently, the sample was subjected to a water flow at 35 ± 1°C for 30 s, followed by drying. The Six stained hair bundles were taken and randomly divided into two groups, three for each group, each was washed using 1 mL 10% SLES, then dried and equilibrated for 24 h in 26 ± 2 ◦C, 60 ± 10% RH. The colour of the hair swatches was measured using the Konica Minolta Spectrophotometer CR400. The treatment of both hair groups is shown in Fig. 6. The measurements in the L, a, and b colour space were recorded. The L, a, and b space consists of three axes: L represents darkness to lightness (with values ranging from 0 to 100), a represents greenness to redness (− 128 to + 127), and b represents blueness to yellowness (-128 to + 127). ∆E represents the difference between two colours in the L, a, and b colour space and is calculated using the following equation:

$$\:\varDelta\:E=\sqrt{({{L}_{1}-{L}_{0})}^{2}+{\left({a}_{1}-{a}_{0}\right)}^{2}+{({b}_{1\:}-{b}_{0})}^{2}}$$

∆E measurements are taken for hair swatches before and after specified treatment-washing cycles. The higher the value, the more the colour difference in swatches due to the treatment-wash cycles. The statistical properties of the sample group and the control group were analysed by TTEST, and the test level was α = 0.05, which was significantly different from the results of the control group.

## Data Availability

Data will be made available on reasonable request. Please contact the email wu1995nan@163.com.
